# Normal hearing function genetics: have you heard all about it? An integrated approach of genome-wide association studies and transcriptome-wide association studies in three Italian cohorts 

**DOI:** 10.3389/fgene.2025.1522338

**Published:** 2025-05-19

**Authors:** Aurora Santin, Giulia Pianigiani, Alessandro Gialluisi, Alessandro Pecori, Beatrice Spedicati, Simona Costanzo, Mariarosaria Persichillo, Francesca Bracone, Giuseppe Giovanni Nardone, Paola Tesolin, Stefania Lenarduzzi, Anna Morgan, Amalia De Curtis, Wouter van der Valk, Francis Rousset, Marta Roccio, Heiko Locher, Licia Iacoviello, Maria Pina Concas, Giorgia Girotto

**Affiliations:** ^1^ Department of Medicine, Surgery and Health Sciences, University of Trieste, Trieste, Italy; ^2^ Institute for Maternal and Child Health - IRCCS “Burlo Garofolo”, Trieste, Italy; ^3^ Department of Epidemiology and Prevention, IRCCS Neuromed, Pozzilli, Italy; ^4^ Department of Medicine and Surgery, LUM University, Casamassima, Italy; ^5^ Neuromed Biobanking Centre, Department of Epidemiology and Prevention, IRCCS Neuromed, Pozzilli, Italy; ^6^ Exobiology Leiden, Department of Otorhinolaryngology and Head and Neck Surgery, Leiden University Medical Center, Leiden, Netherlands; ^7^ The Novo Nordisk Foundation Center for Stem Cell Medicine (reNEW), Leiden University Medical Center, Leiden, Netherlands; ^8^ The Inner Ear and Olfaction Lab, Department of Clinical Neurosciences, Faculty of Medicine, University of Geneva, Geneva, Switzerland; ^9^ Inner Ear Stem Cell Laboratory, Department of Otorhinolaryngology, Head and Neck Surgery, University Hospital Zurich (USZ), Zurich, Switzerland; ^10^ University of Zurich (UZH), Zurich, Switzerland

**Keywords:** Normal Hearing Function, GWAS, ASTN2, SLC1A6, ARF4-AS1

## Abstract

**Introduction:**

Deepening the genetic mechanisms underlying Normal Hearing Function (NHF) has proven challenging, despite extensive efforts through Genome-Wide Association Studies (GWAS).

**Methods:**

NHF was described as a set of nine quantitative traits (i.e., hearing thresholds at 0.25, 0.5, 1, 2, 4, and 8 kHz, and three pure-tone averages of thresholds at low, medium, and high frequencies). For each trait, GWAS analyses were performed on the Moli-sani cohort (n = 1,209); then, replication analyses were conducted on Carlantino (CAR, n = 261) and Val Borbera (VBI, n = 425) cohorts. Expression levels of the most significantly associated genes were assessed employing single-nucleus RNA sequencing data (snRNA-seq) on human fetal and adult inner ear tissues. Finally, for all nine NHF traits, Transcriptome-Wide Association Studies (TWAS) were performed, combining GWAS summary statistics and pre-computed gene expression weights in 12 brain tissues.

**Results:**

GWAS on the Discovery cohort allowed the detection of 667 SNPs spanning 327 protein coding genes at a *p* < 10^−5^, across the nine NHF traits. Two loci with a p < 5 × 10^−8^ were replicated: 1. rs112501869 within *SLC1A6* gene, encoding a brain high-affinity glutamate transporter, reached *p* = 6.21 × 10^−9^ in the 0.25 kHz trait. 2. rs73519456 within *ASTN2* gene, encoding the Astrotactin protein 2, reached genome-wide significance in three NHF traits: 0.5 kHz (*p* = 1.86 × 10^−8^), PTAL (*p* = 9.40 × 10^−9^), and PTAM (*p* = 3.64 × 10^−8^). SnRNA-seq data analyses revealed a peculiar expression of the *ASTN2* gene in the neuronal and dark cells populations, while for *SLC1A6* no significant expression was detected. TWAS analyses detected that the *ARF4-AS1* gene (eQTL: rs1584327) was statistically significant (*p* = 4.49 × 10^−6^) in the hippocampal tissue for the 0.25 kHz trait.

**Conclusion:**

This study took advantage of three Italian cohorts, deeply characterized from a genetic and audiological point of view. Bioinformatics and biostatistics analyses allowed the identification of three novel candidate genes, namely, *SLC1A6, ASTN2,* and *ARF4-AS1*. Functional studies and replication in larger and independent cohorts will be essential to confirm the biological role of these genes in regulating hearing function; however, these results confirm GWAS and TWAS as powerful methods for novel gene discovery, thus paving the way for a deeper understanding of the entangled genetic landscape underlying the auditory system.

## 1 Introduction

Normal Hearing Function (NHF) is a complex sensory process at the base of human communication, sociality, and interaction with the external environment (Dobie and Hemel, 2004). From a genetic viewpoint, NHF is a multifactorial trait, determined by a complex interplay of environmental factors and genetics (Girotto et al., 2011). On the one hand, it is widely recognized that several environmental factors have an impact on hearing ability, such as long-term exposure to loud noises, ototoxic drug intake, or infections (Rosati and Jamesdaniel, 2020). On the other hand, NHF is a complex process tightly regulated by a multitude of different genes (Nishio et al., 2015). Defects in any of these players could potentially lead to hearing loss (HL) (Kremer and Del Castillo, 2022). Moreover, analyzing interindividual variation in hearing traits may help untangle the biological basis of hearing function, leading to greater statistical power than studies comparing a relatively small number of cases vs. controls. A deeper characterization of genes involved in modulating NHF could be a starting point for i) a better understanding of the complex molecular mechanisms regulating the auditory system, and ii) opening novel genetic insights into multifactorial HL.

Therefore, Genome-Wide Association Studies (GWAS) are a powerful strategy for the identification of novel candidate genes underlying NHF (Girotto et al., 2014; Wolber et al., 2014; Vuckovic et al., 2015; Concas et al., 2021). To date, many of these genes have also been successfully validated in functional studies, such as *SIK3* (Wolber et al., 2014), *DCLK1* (Ingham et al., 2020)*, ARSG* and *SLC16A6* (Girotto et al., 2014).

However, despite the efforts made so far, there is still a limited picture of the complex genetic landscape underlying hearing ability.

The integration of GWAS summary statistics with expression quantitative trait loci (eQTLs) data (Nica and Dermitzakis, 2013) is now emerging as a promising approach to pinpoint novel molecular mechanisms underlying complex traits or disorders in a tissue-specific manner. In this light, Transcriptome-Wide Association Studies (TWAS) are a recently developed method that allow estimations of associations between trait-associated genes regulated by Single Nucleotide Polymorphisms (SNPs) identified from GWAS (Mai et al., 2023).

Specifically, TWAS integrate GWAS summary statistics and eQTLs data to test the hypothesis that one or multiple eQTLs can impact the transcriptional activity of a gene; indeed, these genetically regulated gene expression levels can modulate a complex disease risk or phenotypic expression of a multifactorial trait/disorder (Mai et al., 2023). Consequently, TWAS have been gaining attention, as they are able to prioritize potentially causal genes and tissues tagging GWAS hits (Wainberg et al., 2019).

Finally, both for GWAS and TWAS, to obtain solid and reliable results, an in-depth characterization of the phenotype of interest is essential.

Hence, to pinpoint novel candidate genes underlying NHF, this study takes advantage of a combined approach of 1) GWAS, 2) genes expression validation, and 3) TWAS analyses, conducted on three Italian populations: the Moli-sani, Carlantino (CAR), and Val Borbera (VBI) cohorts.

## 2 Materials and methods

### 2.1 Cohorts

Three Italian cohorts, namely, Moli-sani, CAR, and VBI with available audiometric and genotyping data were included in this study.

The Moli-sani study is a general population-based cohort of men and women (aged ≥35 years) residing in the Molise region in the South of Italy (Iacoviello et al., 2012). Between 2005 and 2010, 24,325 subjects (52% women) were recruited from city hall registries (response rate 70%) and underwent a series of assessments, including detailed questionnaires on lifestyle, instrumental assessments, and retrieval of blood and urine samples. A subcohort of the Moli-sani study was recalled between 2017 and 2020 [N = 2,581, (Ruggiero et al., 2022)] and 1,234 participants underwent deep sensorineural assessments, including hearing function.

The CAR (Esko et al., 2012) and VBI (Cocca et al., 2020) cohorts, respectively composed of 311 and 455 individuals, belong to the Italian Network of Genetic Isolates (INGI) project, which is a collaboration project between different research institutions in Italy, referring to a common operational protocol.

### 2.2 Ethical statement

This study complies with the Declaration of Helsinki, and all participating subjects signed informed consent at the time of recruitment.

Specifically, for the Moli-sani study the baseline recruitment was approved by the Ethical Committee of the Catholic University of Rome, Italy, while the follow-up was approved by the Ethics Committee of IRCCS Neuromed, Pozzilli, Italy (ClinicalTrials.gov ID: NCT03119142).

Regarding VBI and CAR, detailed descriptions of these cohorts were reported in Concas et al., 2021. The VBI study was authorized by the Ethical Committee of “San Raffaele” Hospital and Piemonte Region. The CAR study was certified by the Ethics Committee of IRCCS “Burlo Garofolo” (Trieste, Italy), the local administration of Carlantino, and the Health Service of Foggia Province (Italy).

### 2.3 Hearing traits

At enrolment, for each participant, baseline data were examined, and a careful audiological evaluation was performed. Specifically, an audiometric test at six different frequencies (i.e., 0.25, 0.50, 1, 2, 4, and 8 kHz) was conducted for both ears.

Individuals aged less than 18 years, affected by any forms of inherited hearing loss or other diseases potentially leading to hearing defects, as well as subjects exposed to noise or ototoxic medications, were excluded from this study (Supplementary Table S1).

NHF was described as a set of nine quantitative traits, as previously reported in the literature (Girotto et al., 2011; Concas et al., 2021). Specifically, the following nine parameters were analyzed: i) the hearing thresholds at the six frequencies listed above, and ii) the three Pure Tone Averages (PTAs) at low (PTAL: 0.25, 0.50, 1 kHz), medium (PTAM: 0.50, 1, 2 kHz), and high frequencies (PTAH: 4, 8 kHz), that by analyzing partially overlapping information, allow a deeper evaluation of hearing ability. Since some sounds are typically associated to specific frequencies while others include a fundamental frequency and its harmonics, there are multiple genes possibly regulating hearing ability. Hence, these PTAs provide a more comprehensive assessment of hearing function.

Considering that men and women display different audiometric profiles with respect to their age, each hearing trait was corrected for age and sex; residuals derived from this regression were cleaned from outliers (mean ± three standard deviations) and, considering their skewed distribution, rank-based inverse normalization was performed.

### 2.4 Genotyping, quality control, and imputation

For each individual in the three cohorts, DNA was extracted from blood samples and genotyped. In particular, genotyping was performed with the following platforms: i) Illumina Infinium Global Screening Array v.1 (642K) and Illumina Infinium Global Screening Array v.3 (654K) beadchips for Moli-sani cohort, ii) Illumina 370K beadchip for CAR, iii) Illumina 370K and 700K beadchips for VBI (Illumina Inc., San Diego, CA, United States).

Standard quality control (QC) was conducted both for samples and for the SNP-arrays data. Concerning the samples, the following parameters have been applied: sample call-rate ≥0.95 and cross-check between referred and genetically inferred sex for each participant (Supplementary Table S1); regarding SNP-array data the following criteria have been checked: call rate ≥0.95, Hardy–Weinberg Equilibrium *p*-value > 1 × 10^−6^, and minor allele frequency (MAF) ≥0.01. All of these steps were performed in each cohort separately.

At the end of these QC passages, the cohorts were composed by the following number of individuals: i) 1,209 participants for the Moli-sani cohort, ii) 261 subjects for the CAR cohort, and iii) 425 individuals for the VBI cohort.

The following number of SNPs have been included after the QC steps: i) 735,528 SNPs for the Moli-sani cohort, ii) 309,418 SNPs for the CAR cohort, and iii) 329,278 SNPs for the VBI cohort. Genotypes were called with Illumina GenomeStudio, referred to the forward strand, and reported with the coordinates of the 1000 Genomes Project build 37 (Auton et al., 2015). Imputation was conducted on the Italian Genome Reference Panel (Cocca et al., 2020) with IMPUTE2 software (Howie et al., 2012), and variants with an INFO score <0.4 were discarded. Overall, a total number of i) 11,942,356 SNPs for the Moli-sani cohort, ii) 2,192,965 SNPs for the CAR cohort, and iii) 2,186,136 for the VBI cohort have been included for GWAS analyses.

### 2.5 Genome-wide association study, replication and meta-analyses

GWAS for the nine traits described above were carried out singularly on each cohort employing a linear mixed model regression, assuming an additive genetic model. Since the traits were already adjusted for age and sex, the following covariates were added: the genomic kinship matrix, to address the relatedness of samples in all three cohorts, and the first ten Principal Components (PCs). PCs were computed from genetic data, using PLINK software v1.07 [http://pngu.mgh.harvard.edu/purcell/plink/, accessed on 26 February 2025 (Purcell et al., 2007)]. The kinship matrix, calculated using GEMMA for Moli-sani cohort and GenABEL (Aulchenko et al., 2007) for CAR and VBI, was employed as a random effect.

Association analyses for the Moli-sani cohort were performed with the GEMMA v0.98 software (Zhou and Stephens, 2012), while VBI and CAR were previously analyzed in R (www.r-project.org, accessed on 30 October 2024), using the GRAMMAR-γ method (Concas et al., 2021).

Moli-sani was considered as the “Discovery cohort”, while CAR, and VBI as the “Replication cohorts”. In this light, all the SNPs deriving from the GWAS on the Discovery cohort with a *p* < 1 × 10^−5^ have been considered for the replication analysis. To be considered a replica, the association in each of the Replication cohorts had to present the concordant direction of effect, and *p* ≤ 0.05 in at least one Replication cohorts.

To obtain an overall summary statistic, GWAS results on the Moli-sani, CAR, and VBI cohorts were combined through a fixed-effect sample size based meta-analysis using METAL software [version released on 2011-03-25, (Willer et al., 2010)], thus reaching an overall sample of 1895 individuals. Genome-wide significance was set to *p* < 5 × 10^−8^. SNPs were annotated with the Variant Effect Predictor tool (VEP, https://www.ensembl.org/info/docs/tools/vep/index.html, accessed on the 26 March 2025). Long non-coding RNAs genes or genes with unknown function identified with LOC and FAM symbols or pseudogenes were excluded.

### 2.6 Validation of genes expression

For the genes most-significantly associated to NHF traits (selected according to their *p*-value and biological significance in relation to NHF), expression data were extracted from the single-nucleus atlas of human fetal (fetal age week 7.5, and 9.2) and adult (from a 47-year-old donor) inner ear tissues published by van der Valk et al., 2023). Violin plots were constructed using Seurat (Satija et al., 2015) and ggplot2 (https://ggplot2.tidyverse.org/, accessed on the 26 March 2025) to display the gene expression among the following inner ear cell types: mesenchymal cells, epithelial cells, hair cells, neurons, glial cells, cycling cells, endothelial cells, macrophages, and melanocytes.

### 2.7 TWAS analyses

GWAS Meta-analyses summary statistics of the combined sample were properly formatted for TWAS analyses, employing the LD Score Regression (LDSC, v1.0.1) software. This step allowed correction of inflated test statistics from confounding bias and polygenicity, thus generating a sumstat-formatted file corrected from the inflation values caused by GWAS (Bulik-Sullivan et al., 2015).

TWAS analyses were conducted with FUSION software, following TWAS FUSION protocol with default settings (http://gusevlab.org/projects/fusion/, accessed on the 26 March 2025), that allows integration of GWAS summary statistics and pre-computed gene expression weights, with default settings.

Considering that currently, no pre-computed gene expression weights are available for inner ear tissues, brain tissues panels were considered for TWAS analyses.

In particular, twelve SNP-weights panels (i.e., Brain Amygdala, Brain Anterior cingulate cortex (BA24), Brain Caudate (basal ganglia), Brain Cerebellar Hemisphere, Brain Cerebellum, Brain Cortex, Brain Frontal Cortex (BA9), Brain Hippocampus, Brain Hypothalamus, Brain Nucleus accumbens (basal ganglia), Brain Putamen (basal ganglia), Brain Substantia nigra) from postmortem brain tissues were downloaded from TWAS FUSION website (http://gusevlab.org/projects/fusion/#reference-functional-data, a accessed on the 26 March 2025). TWAS were carried out for all nine NHF traits firstly in each of the SNP-weights brain panels mentioned above, and then combing all the panels together. All *p*-values of each performed TWAS were subjected to multiple comparison testing using Bonferroni correction (0.05/number of genes in each tested panel) to evaluate associations between genes located near GWAS signals and NHF traits. The number of genes present in each considered panel are reported in Supplementary Table S2.

### 2.8 Colocalization analysis

To evaluate the reliability of TWAS results, a colocalization analysis (Giambartolomei et al., 2014) was performed for all genes at transcriptome-wide significance. This is a Bayesian approach that allows estimates of five posterior probabilities (PP): 1) no association with either a GWAS signal or eQTL (PP0), 2) association with a GWAS signal only (PP1), 3) association with a signal eQTL only (PP2), 4) association with a GWAS signal and eQTL with two independent SNPs (PP3), 5) and association with a GWAS signal and eQTL having one shared SNP (PP4).

In this study, default priors in which a random variant in the region is associated with either a GWAS or an eQTL individually were fixed at *p* = 1 × 10^−04^, while the prior probability that a random variant is causal to both GWAS and eQTLs was set at *p* = 1 × 10^−05^, as previously reported (Kia et al., 2021). In particular, the following combination cutoffs to provide evidence of colocalization were employed: PP4 ≥ 0.75, PP3+PP4≥0.9, PP4/PP3≥ 3 (Kia et al., 2021; Pan et al., 2022).

## 3 Results

### 3.1 Cohorts’ description and study workflow

In this study, three Italian cohorts were included for GWAS analysis. In detail, the Moli-sani cohort (Discovery cohort) comprised 1,209 individuals, with a mean age of 64 years, of whom 56% were women. The CAR cohort was composed of 261 participants with an average age of 53.2 years; 57% of them were women. The VBI cohort included 425 individuals (58% female) with an average age of 58.7 years. All participants of the three cohorts underwent a careful audiological evaluation; results of the audiometric assessment are summarized in Table 1.

**TABLE 1:** Cohorts’ characteristics.

Cohort	N	N Men; N Women	Age	0.25 kHz	0.50 kHz	1 kHz	2 kHz	4 kHz	8 kHz	PTAL	PTAM	PTAH
Moli-sani	1,209	531 (44%); 678 (56%)	46–89 (64.3 ± 9.32)	0–90 (16.3 ± 9.92)	0-90 (16.3 ± 10.40)	0–90 (16.9 ± 12.5)	0–90 (18.7 ± 16.4)	0–90 (31.3 ± 21.1)	0–90 (37.7 ± 25.6)	0–90 (17.1 ± 10.2)	0–90 (18.0 ± 12.0)	0–90 (35.3 ± 21.9)
CAR	261	113 (43%); 148 (57%)	18–89 (53.2 ± 18.0)	0–95 (19.0 ± 11.8)	0–100 (16.4 ± 12.7)	0–115 (15.7 ± 14.8)	0–115 (17.3 ± 18.4)	0–130 (26.5 ± 24.0)	0–130 (31.84 ± 25.30)	0–105 (17.4 ± 12.6)	0–110 (16.9 ± 14.6)	0–130 (29.8 ± 24.1)
VBI	425	180 (43%); 245 (58%)	25–90 (58.7 ± 15.3)	5–75 (17.2 ± 8.46)	5–70 (18.47 ± 9.69)	10–95 (20.52 ± 11.55)	5–90 (22.64 ± 16.3)	5–100 (30.54 ± 21.27)	10–125 (34.94 ± 25.46)	5–58.33 (18.96 ± 8.9)	8.33–70 (20.94 ± 11.37)	2.5–112.5 (33.18 ± 22.69)

This table shows minimal demographic and audiometric data (relative to 0.25–8.0 kHz, PTAL, PTAM, PTAH) of the analyzed cohorts. Data in columns 4-13 are reported as minimum-maximum (mean, standard deviation). Cohort, name of the analyzed cohorts; N, Total number of individuals for each cohort; N Men, N Women, Total number of men and women for each cohort; 0.25–8 kHz, Audiometric values from low to high frequencies; PTAL, PTAM, PTAH, Pure Tone Averages are described as the averages of audiometric data at low (PTAL), medium (PTAM), and high frequencies (PTAH).

To pursue the aim of this study, the following steps have been performed:

1. Audiometric and genotyping data of the Discovery cohort have been combined in nine GWAS analyses, one for each NHF trait.
2. All the association signals with a *p* < 1 × 10^−5^ resulting from the GWAS on the Discovery cohort have been considered for the replication analysis on CAR and VBI cohorts, and then combined together in GWAS Meta-analyses.
3. The expression of the most significantly associated genes across the NHF analyzed traits was evaluated employing human fetal and adult snRNA-seq data (van der Valk et al., 2023).
4. GWAS summary statistics were then integrated with brain eQTL data to detect potentially causal genes and tissues underlying GWAS hits.

**FIGURE 1:**
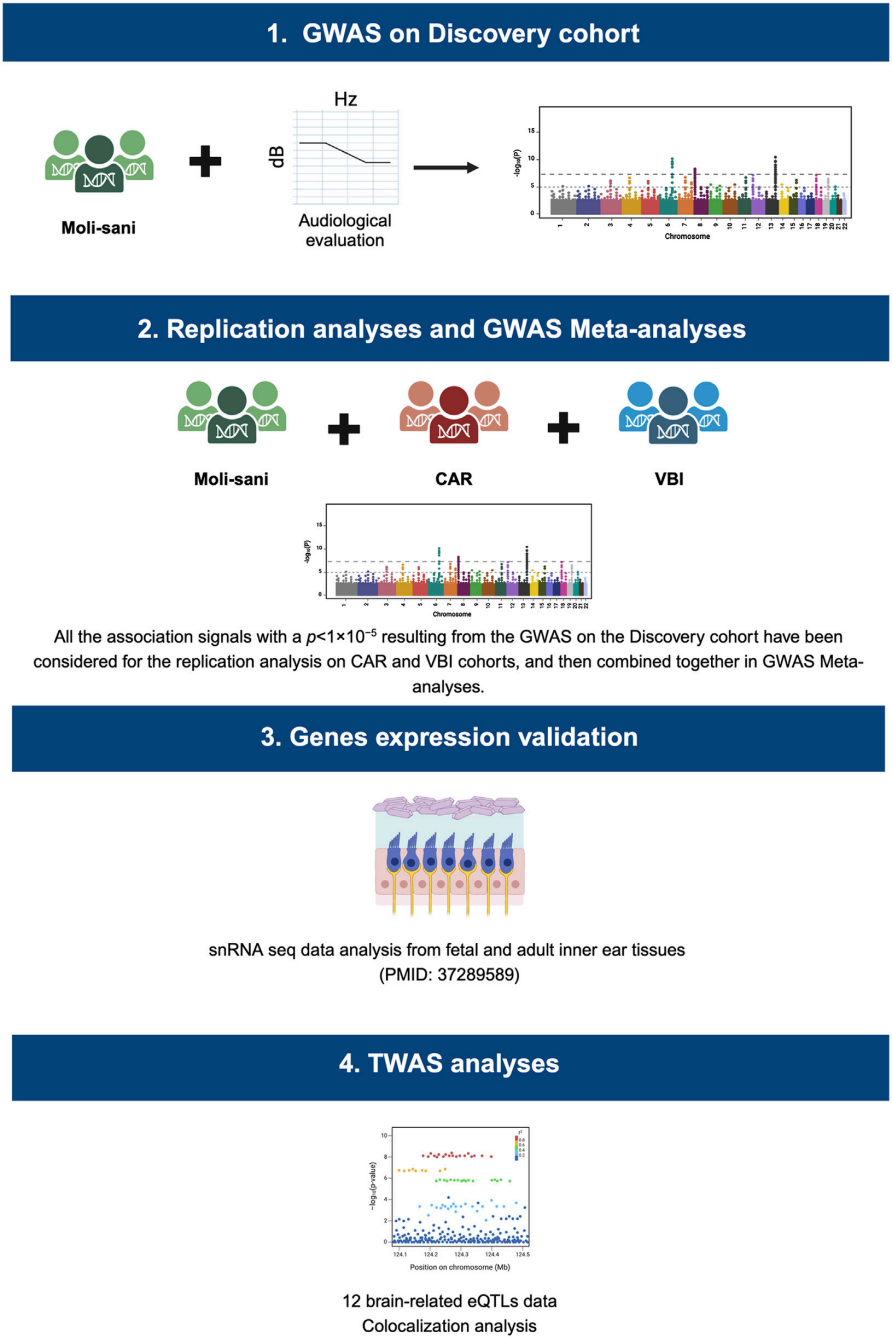
Study workflow. This image displays the main steps of this study. Specifically, GWAS analysis, one for each NHF trait, have been performed on the Discovery cohort (Moli-sani). All the association signals with a *p* < 1 × 10^−5^ have been considered for the replication analysis on CAR and VBI cohorts, and then combined. The expression of the most significantly associated genes across the NHF analyzed traits was validated employing human fetal and adult snRNA-seq data (van der Valk et al., 2023). GWAS Meta-analyses summary statistics were then integrated with brain eQTL data in a TWAS analysis. CAR, Carlantino; VBI, Val Borbera.

Complete study workflow is represented in Figure 1.

### 3.2 Association analyses results

GWAS on the Discovery cohort detected 667 SNPs spanning 327 different protein coding genes at *p* < 10^−5^, across the nine NHF traits. For each NHF trait, Manhattan and QQ plots are reported in Supplementary Figures S1,S2, respectively.

All the association signals resulting from the GWAS on the Discovery cohort with a p < 1 × 10^−5^ have been considered for the replication analysis on CAR and VBI cohorts.

At the nominal significance (p < 0.05) and with concordant effect (i.e., beta coefficient), a total of 65 association signals within 18 protein coding genes in the CAR cohort, and 60 association signals within 35 protein coding genes in the VBI cohort have been replicated across the nine hearing traits.

Overall, two loci with p < 5 × 10^−8^ were identified in the GWAS Meta-analyses (Moli-sani, CAR, VBI) as reported in Table 2 and Supplementary Figure S3 the Manhattan plots of the GWAS Meta-analyses are reported.

**TABLE 2:** Main results from GWAS Meta-analyses selected according to significance threshold.

						Moli-sani				CAR				VBI				Moli-sani - CAR - VBI	
Trait	Top SNP	Chr	Ps	Nearest Gene	Effect allele/Other allele	AF	beta	StdErr	p-value	AF	beta	StdErr	p-value	AF	beta	StdErr	p-value	Zscore	p-value
0.25 kHz	rs112501869	19	15079695	*SLC1A6*	T/A	0.02	8.33	1.45	1.8 × 10^−08^	0.02	0.07	0.27	5.9 × 10^−02^	0.02	0.08	0.03	4.4 × 10^−02^	5.81	6.21 × 10^−09^
0.5 kHz	rs73519456	9	119538296	*ASTN2*	T/G	0.02	7.24	1.40	3.8 × 10^−07^	0.03	0.04	0.08	6.2 × 10^−01^	0.01	0.17	0.06	2.7 × 10^−03^	5.63	1.86 × 10^−09^
PTAL							6.38	1.30	1.3 × 10^−06^		0.05	0.06	3.9 × 10^−01^		0.17	0.05	7.2 × 10^−04^	5.74	9.40 × 10^−09^
PTAM							6.78	1.48	5.9 × 10^−06^		0.07	0.07	3.3 × 10^−01^		0.18	0.05	9.7 × 10^−04^	5.51	3.64 × 10^−08^

This table shows the GWAS results for the Moli-sani cohort, the replication analyses results on CAR, VBI cohorts and results of the GWAS Meta-analyses on the three cohorts reaching significant and suggestive *p*-values across the nine NHF traits. Trait, analyzed hearing trait; Top SNP, most significant SNP identified; Chr, chromosome; Ps, position of the variant (build hg19); Nearest gene, name of the nearest gene identified with VEP; Effect allele/Other allele, effect allele/other allele; AF, frequency of the effect allele; beta, beta effect from GWAS analyses; StdErr, standard error of beta; Zscore, effect from GWAS Meta-analyses. All the detailed variants have an INFO score >0.7 (for further details, please see the Supplementary Tables S3–S11).

In particular, for the 0.25 kHz trait, an intronic SNP, rs112501869, reached a *p* = 6.21 × 10^−9^. This SNP maps within the *SLC1A6* gene, encoding a high-affinity glutamate transporter mainly expressed in Purkinje cells and the regional plot is represented in Supplementary Figure S4A (Wilmsdorff et al., 2013).

Further, the other intronic SNP, namely, rs73519456, reached genome-wide significance in three NHF traits, respectively 0.5 kHz (*p* = 1.86 × 10^−8^), PTAL (*p* = 9.40 × 10^−9^), and PTAM (*p* = 3.64 × 10^−8^) (for the regional plots please see Supplementary Figures S4B–D). This SNP is located within *ASTN2* gene, encoding the Astrotactin protein 2, and mainly expressed in the cerebellum (Behesti et al., 2018). The role of this gene is not fully elucidated yet; however, it was recently demonstrated that *ASTN2* is involved in glial-guided neuronal migration, and modulation of dendritic spine strength (Wilson et al., 2010; Behesti et al., 2018).

Complete results of GWAS analysis on the Discovery cohort, replication analyses on CAR and VBI cohorts, and GWAS Meta-analyses on the three cohorts’ data (i.e., Moli-sani, CAR, and VBI) are reported in Supplementary Tables S3–S11. VEP annotation is detailed in Supplementary Table S12.

### 3.3 Genes expression evaluation

**FIGURE 2:**
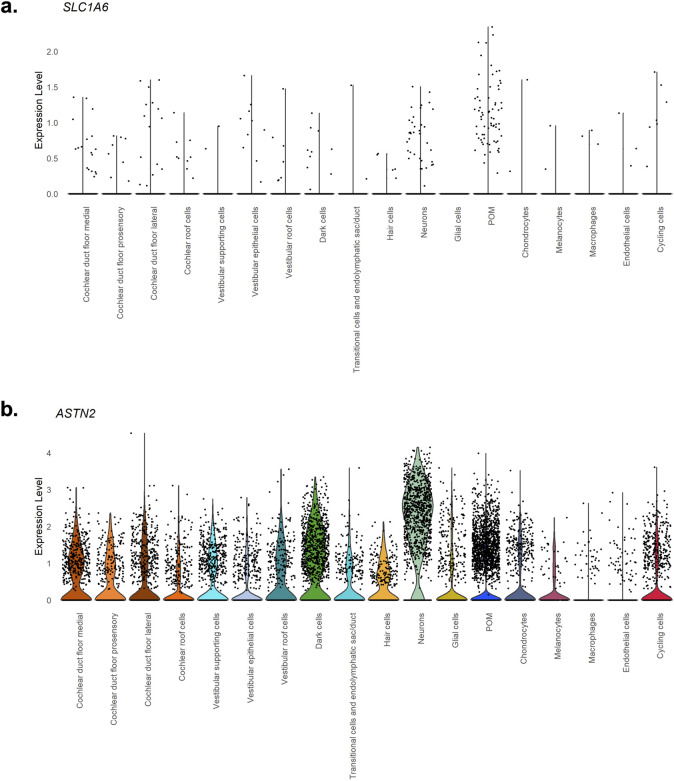
Violin plots showing expression levels of genes prioritized in the GWAS Meta-analyses. The violin plots report the expression levels. **(a)** *SLC1A6*, **(b)** *ASTN2* genes in human inner ear tissues, extracted from snRNA-seq data (van der Valk et al., 2023). In the x-axis inner ear cell types are detailed, and in the y-axis, expression values are reported.

To further characterize the potential biological role in NHF of the genome-wide significant GWAS Meta-analyses hits, namely, a) *SLC1A6* gene and b) *ASTN2* gene, the expression patterns of these two genes were extracted from snRNA-seq data in human inner ear tissues (van der Valk et al., 2023). Results are shown in Figure 2.

In particular, according to the expression values reported in Figure 2, *ASTN2* is expressed in the neuronal and dark cells populations. Regarding *SLC1A6* no significant expression levels are detected in the inner ear.

### 3.4 TWAS and colocalization analyses

**FIGURE 3:**
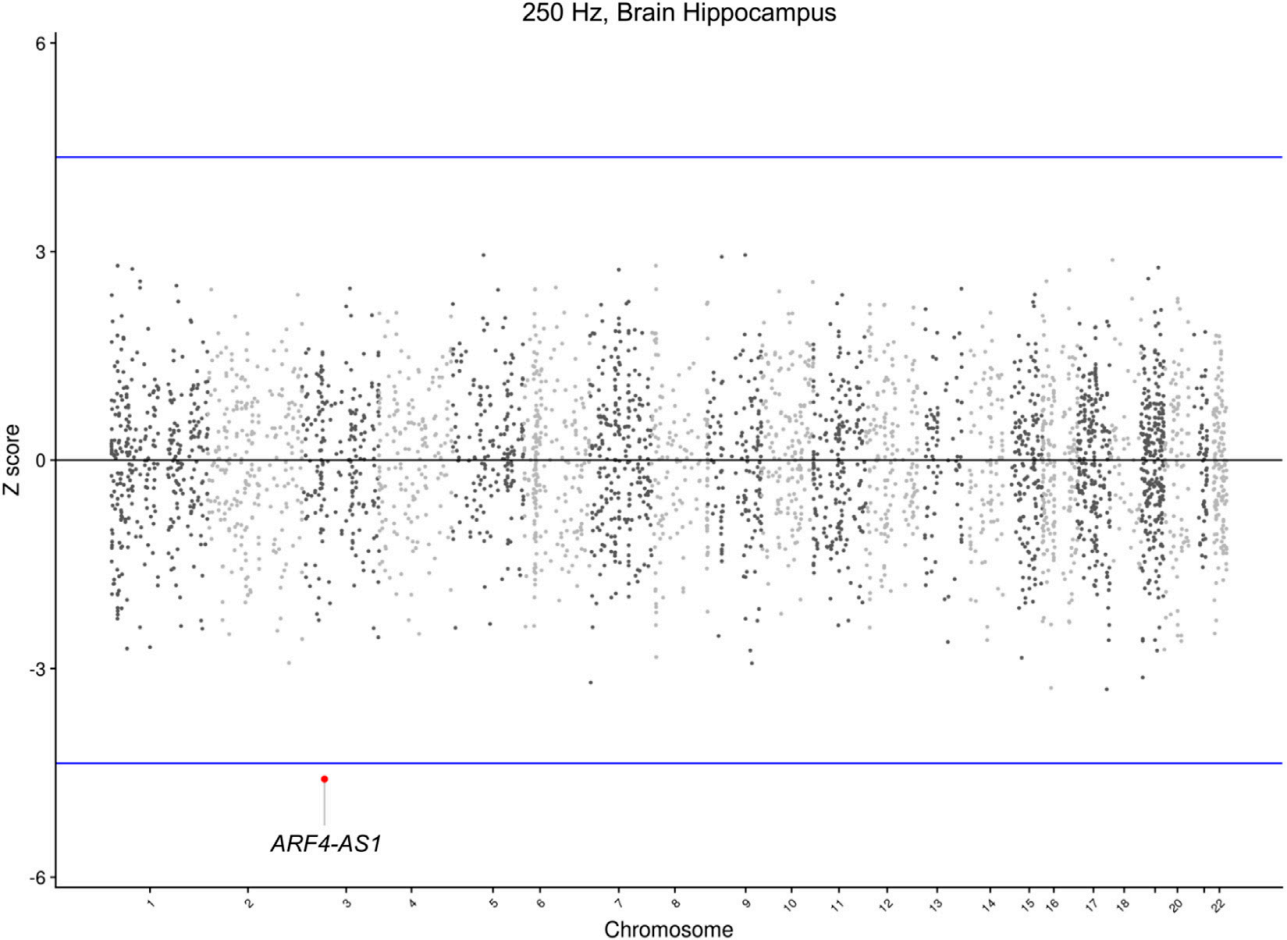
Manhattan plot of TWAS results at 0.25 kHz in Brain Hippocampus tissue. This Manhattan plot displays the Z-score (y-axis) for the TWAS on the 0.25 kHz trait and the Brain Hippocampus tissue panel. The blue horizontal lines represent the Bonferroni-corrected significance threshold. Significant genes were highlighted with their gene symbol.

To further identify novel candidate genes for NHF, TWAS analyses were conducted. In particular, TWAS were carried out combining GWAS Meta-analyses summary statistics and pre-computed gene expression weights in 12 brain tissues, as detailed in the Materials and Methods section. TWAS results analyses detected that, after Bonferroni correction, a long non-coding gene, namely, *ARF4-AS1* (Ensembl ID: ENSG00000272146.5, eQTL: rs1584327), was statistically significant (p = 4.49 × 10^−6^) in the hippocampal tissue for the 0.25 kHz trait (Figure 3; Table 3).

**TABLE 3:** TWAS results for 0.25 kHz trait in GTeX v.8 Brain Hippocampus.

ID	CHR:POS	Best. GWAS.ID	Best. GWAS.Z	EQTL.ID	EQTL.R2	EQTL.Z	EQTL.GWAS.Z	NSNP	Model	MODELCV.R2	MODELCV.PV	TWAS.Z	TWAS.P
ENSG00000272146.5	3:57600058–57600059	rs268776	−4.26	rs1584327	0.03	4.33	−4.059	330	enet	0.056	0.0013	−4.59	4.49 × 10^−6^

ID, Gene identifier; CHR:POS, Chromosome, gene start and end position; BEST.GWAS.ID, rsID of the most significant GWAS Meta-analysis SNP in the locus; BEST.GWAS.Z, Z-score of the most significant GWAS Meta-analysis SNP in the locus; EQTL.ID, rs ID of the best eQTL in the locus; EQTL.R2, cross-validation R2 of the best eQTL in the locus; EQTL.Z, Z-score of the best eQTL in the locus; EQTL.GWAS.Z, GWAS Meta-analysis Z-score for this eQTL; NSNP, Number of SNPs in the locus; MODEL, best performing model; MODELCV.R2, cross-validation R2 of the best performing model; MODELCV.PV, cross-validation P-value of the best performing model; TWAS.Z, TWAS Z-score; TWAS.P, TWAS P-value.

Considering that 48% of genetic variants act as an eQTL for at least one gene (Cano-Gamez and Trynka, 2020), it is possible that GWAS and eQTL signals could overlap by chance; therefore, a colocalization analysis was performed.

Setting the prior probability that the detected variant is associated to both NHF and gene expression regulation in the hippocampus equal to 1.0 × 10^−6^, COLOC analysis results (PP4 = 0.8, PP3 + PP4 = 0.9, and PP4/PP3 = 27) support a possible causal role of *ARF4-AS1* in the transcriptomic modulation of NHF at 0.25 kHz (Table 4).

**TABLE 4:** Colocalization analysis of TWAS results for 0.25 kHz trait in GTeX v.8 Brain Hippocampus tissue.

ID	CHR:POS	TWAS.P	COLOC.PP0	COLOC.PP1	COLOC.PP2	COLOC.PP3	COLOC.PP4	PP3 + PP4	PP4/PP3
ENSG00000272146.5	3:57600058–57600059	4.49E-06	0.044	0.022	0.064	0.031	0.839	0.87	27.06

ID, Gene identifier; CHR:POS, Chromosome, gene start and end position; TWAS.P, TWAS P-value.COLOC.PP0, posterior probability of SNP association with either a GWAS signal or eQTL.COLOC.PP1, posterior probability of SNP association a GWAS signal only; COLOC.PP2, posterior probability of SNP association with a signal eQTL only; COLOC.PP3, posterior probability of SNP association with a GWAS signal and eQTL with two independent SNPs; COLOC.PP4, posterior probability of SNP association with a GWAS signal and eQTL having one shared SNP; PP3 + PP4, cut-off threshold that if over 0.9 provides evidence of colocalization; PP4/PP3, cut-off threshold that if over or equal to 3.0 provides evidence of colocalization.

Finally, *ARF4-AS1* gene expression was evaluated in the snRNA-seq data (van der Valk et al., 2023); no significant expression levels were detected in the inner ear (Supplementary Figure S5).

## 4 Discussion

Hearing is a tightly regulated complex process that converts sound waves into mechanical and then electrical signals that can be interpreted and processed by the brain (Fuchs and Tucker, 2015). Any alterations to this sequence of events could impact proper hearing ability (Prasad and Bondy, 2020). Hearing defects comprise a considerable number of disorders, clinically and genetically heterogeneous (Kremer and Del Castillo, 2022); therefore, considering the number of structures and genes involved in hearing function, detangling the complex molecular mechanisms of the auditory system could open intriguing genetic insights into HL complex forms. Unraveling the genetic architecture of NHF has proven challenging; indeed, despite many studies having been conducted, full knowledge of NHF genetics has not yet been reached.

In this study, in order to identify novel candidate genes involved in NHF modulation, three Italian cohorts (i.e., Moli-sani, CAR and VBI) deeply characterized from an audiological point of view have been analyzed. Here, for the first time, the Moli-sani cohort, selected in the framework of the Moli-sani study (Iacoviello et al., 2012), has been investigated in relation to hearing ability through a GWAS approach; regarding CAR and VBI cohorts, these are two genetically isolated Italian populations, belonging to the INGI project, that have been already studied for the identification of novel candidate genes potentially involved in NHF in a previous GWAS (Concas et al., 2021).

In this work the following steps were conducted: firstly, GWAS analyses were performed on the Discovery cohort, allowing the identification of 667 SNPs spanning 327 different protein coding genes at a *p* < 10^−5^ across the nine NHF traits. Secondly, replication analyses detected 65 SNPs within 18 protein coding genes in the CAR cohort, and 60 SNPs within 35 protein coding genes in the VBI cohort, reaching nominal significance (*p* < 0.05) and with concordant effect.

Finally, GWAS Meta-analyses were conducted, highlighting 539 SNPs spanning 203 genes, reaching *p* < 1.0 × 10^−5^. Notably, the variants that reached genome-wide significance were:

i) rs112501869 mapping within the *SLC1A6* gene (*p* = 6.21 × 10^−9^) in the 0.25Hz trait, and
ii) rs73519456, located within *ASTN2* gene, in three NHF traits (i.e., 0.5 kHz with a *p* = 1.86 × 10^−8^, PTAL with a *p* = 9.40 × 10^−9^, and PTAM with a *p* = 3.64 × 10^−8^).

These variants reached suggestive *p*-values (*p* < 1 × 10^−5^) in the Discovery cohort and nominal significance in the VBI cohort, but no significance was found in the CAR cohort, even if their effects were concordant.

This could be ascribed to the limited sample size of the CAR cohort that may have influenced the statistical power of the analyses, thus hampering the replication of these results. Further investigations in independent cohorts with higher sample sizes will be needed to give relevance to these findings.

However, overall GWAS Meta-analyses results revealed two promising NHF candidate genes, namely, *SLC1A6* and *ASTN2.*

The *SLC1A6* gene encodes a member of the high affinity glutamate transporter family (EAATs) that regulates glutamate concentration at the synaptic cleft (Wilmsdorff et al., 2013). As of now, this study represents the first report describing *SLC1A6* in hearing function; however, other members of the same transporter family, such as the *SLC16A6* gene (Girotto et al., 2014), have previously been linked to auditory processes within the inner ear.

Regarding the inner ear compartment, the snRNA-seq data published by van der Valk et al. revealed no detectable expression of *SLC1A6* gene expression in human inner ear cell lines (van der Valk et al., 2023). Given its known neuronal expression profile, this suggests that *SLC1A6* may exert its role in auditory function at the brain level. Indeed, EAATs are responsible for extracellular glutamate reuptake, thus preventing glutamate excitotoxicity events (Pérez-Mato et al., 2019) that have been associated with several pathological conditions (Lau and Tymianski, 2010), including HL (Tadros et al., 2007).

Supporting this hypothesis, a gene of the same family of *SLC1A6*, namely, *Slc1a3*, was found to be upregulated in the auditory midbrain of aged HL mice models, suggesting the existence of a cellular compensatory mechanism aimed at mitigating excessive glutamate release during the aging process (Tadros et al., 2007). Overall, the present findings could raise the hypothesis that also *SLC1A6* may contribute to the central auditory homeostasis maintenance by limiting glutamate-mediated neurotoxicity. This could open novel avenues for deepening its role in auditory signal regulation and age-related hearing function safeguarding.

Concerning *ASTN2* gene, an extensive review of the literature revealed that it has been also associated with self-reported hearing difficulty in a previous GWAS study conducted on UKBiobank data (top SNP: rs10739473, p = 2.26 × 10^−8^) (Liu et al., 2021). Through the employment of LinDA genome browser (http://linda.irgb.cnr.it/index.html), it was possible to observe that the *ASTN2* variant detected in this study (i.e., rs73519456) and the variant highlighted in the aforementioned GWAS study on the UK Biobank cohort are in a high Linkage Disequilibrium (LD) (r^2^ = 1) in European populations. These findings may suggest that these two variants, being in LD, may serve as proxies for one another, implying that both variants could capture the association signal attributable to the same underlying causal locus. Overall, these results provide further evidence for the involvement of *ASTN2* in hearing function, highlighting the need for additional follow-up studies and defining this gene as a promising candidate in the modulation of hearing.

Notably, the same variant detected in this study (i.e., rs73519456) has also been reported to be associated with middle-ear disorders, including tympanic membrane, eustachian tube disorders, and tympanic membrane perforation, as documented in the PheWeb catalog (https://pheweb.org/UKB-TOPMed/variant/9:116776017-G-T). Considering that it is recognized that common middle ear disorders often can result in conductive hearing loss (Zhao et al., 2009), these findings suggest that *ASTN2* may represent a novel candidate involved in the underlying mechanisms of such conditions.

The analyses of the human inner ear snRNA-seq data (van der Valk et al., 2023) revealed its expression in the i) dark and ii) neuronal cells populations. Dark cells play a key role in maintaining the endolymphatic potassium-sodium content, and their the degeneration has been associated with several HL conditions, such as Waardenburg syndrome and Méniére’s disease (Kaya et al., 2017). Neuronal cells, responsible for transmitting auditory signals to the brain, are particularly susceptible to degenerative processes during aging (Pavlinkova, 2020), thus influencing proper hearing function. Hence, while *ASTN2*’s role in inner ear neurons remains to be characterized, its known expression in these cell types suggests a potential dual function in modulating auditory processes at both ear and brain levels.

Although functional validation of the role of these genes is still required, these findings confirm GWAS as a powerful method in detecting novel candidates underlying NHF, thereby advancing the understanding of the entangled genomic architecture underlying the hearing process.

To further identify novel possible NHF-associated genes, in this work GWAS Meta-analyses summary statistics have been integrated with eQTL data, thus performing, to the best of our knowledge, the first TWAS analysis on NHF. However, as of now, eQTL data for the inner ear are unavailable on the GTEx database; future implementation of inner ear tissue expression panels would undoubtedly provide the most suitable data source for a deeper comprehension of the genetically regulated gene expression mechanisms underlying hearing function. Considering the absence of these data, the most appropriate strategy was to select the most relevant tissue panels for hearing function regulation from the available options, specifically those related to the brain; consequently, the TWAS analyses were performed on a panel of 12 brain tissues. However, even if this could be perceived as a limitation of this study, it should be considered that all the brain selected tissues are known to be involved in auditory signals processing and integration, thus highlighting the relevance of the central nervous system in regulating NHF.

In the 0.25 kHz trait, TWAS analyses revealed that the *ARF4-AS1* gene was statistically significant in the hippocampal tissue, confirmed by the colocalization analysis.

In-depth research of the literature revealed that the hippocampus has a key role in auditory signals integration and processing. Specifically, it has been reported that the volume of hippocampus correlates positively with auditory stimulation (Billig et al., 2022), and, in accordance with this evidence, patients affected by hearing impairment display significantly smaller hippocampal volumes compared with controls (Uchida et al., 2018).

To date, no studies have reported a link between the *ARF4-AS1* and hearing function. Nevertheless, it could be speculated that this gene could be involved in the regulation of its sense gene expression, *ARF4*. The *ARF4* gene encodes the GTPase ADP ribosylation factor 4 that modulates dendritic spine development in the dentate gyrus of hippocampus (Jain et al., 2012). Although no direct association between *ARF4* and the auditory system has been reported thus far, a comprehensive literature review revealed that *ARF4* is expressed in the murine cochlear epithelium (Hoa et al., 2021), and also reported in the gEAR database (https://umgear.org/expression.html?gene_symbol=arf4&is_multigene=0&layout_id=f64f9c22, accessed on the 26 March 2025). Further studies with *in vitro* and *in vivo* models will be needed to validate the relevance of these findings.

However, it is relevant to highlight that in this study TWAS analyses failed in capturing GWAS analyses’ significant associations. Nevertheless, according to the literature, there are some reports describing possible discrepancies between GWAS and TWAS findings, even when conducted in the same population and for the same trait. Indeed, several factors could be involved in determining this issue, including:

• Effects of GWAS variants: it is possible that not all GWAS SNPs could influence gene expression levels. In fact, not all GWAS variants are eQTLs. Indeed, some SNPs may only impact protein structure, or epigenetic regulations, which are not evaluated by TWAS (Fabo and Khavari, 2023).
• Impact of the LD: TWAS may prioritize a different gene in LD as the most likely causal candidate, compared to GWAS. In fact, TWAS may take into account the LD in giving the results; in this case, a GWAS signal could be removed if the association is primarily driven by another gene (Li and Ritchie, 2021).
• Statistical power of TWAS: eQTL datasets used in TWAS could have limited sample sizes compared to GWAS (e.g., eQTL panels include hundreds of samples, while GWAS studies often analyze thousands of them). In this case, if the eQTL dataset lacks sufficient power, TWAS may fail to detect an association, even when the gene is significantly associated in the GWAS analysis.

To deepen the causal reason for these differences between GWAS and TWAS findings, it could be useful to perform a functional evaluation of GWAS variants to shed light on their real impact on the encoded protein and to pinpoint potential regulatory effects.

In conclusion, this study takes advantage of three accurately characterized Italian cohorts, describing for the first time the Moli-sani cohort in relation to hearing phenotypes. GWAS analyses allowed the identification of two genome-wide significant novel candidate genes:

i) *SLC1A6* gene that could be involved in the regulation of auditory signals at the brain level.
ii) *ASTN2* gene, which considering its peculiar expression in neuronal and dark cells, could play a dual role in regulating hearing function, both at the ear and brain level.

Finally, this is the first study performing a TWAS on NHF, leading to the identification of *ARF4-AS1* gene, whose regulatory role could open novel insights into the brain processing of hearing signals.

Overall, these findings confirm the potentiality of biostatistics/bioinformatic methods in the discovery of novel genes and variants underlying complex traits and disorders. Further characterization of these newly identified genes with functional studies will be needed. Moreover, future implementation of colocalization analyses between the variants detected in this study and HL association signals from large-scale studies will be essential to elucidate potential shared genetic mechanisms between NHF and HL. This, in turn, could provide novel insights into the molecular mechanisms underlying the auditory system, thereby offering new perspectives on the etiopathogenesis of multifactorial HL and paving the way for the development of novel therapeutic targets for HL patients.

## Data Availability

The datasets presented in this study can be found in online repositories. The names of the repository/repositories and accession number(s) can be found below: https://www.ebi.ac.uk/eva/, PRJEB79914; https://www.ebi.ac.uk/eva/, PRJEB33648.
