# Supplementary material for: Normal hearing function genetics: have you heard all about it? An integrated approach of genome-wide association studies and transcriptome-wide association studies in three Italian cohorts
Source: Front Genet. 2025 May 19;16:1522338. doi: 10.3389/fgene.2025.1522338 (PMC12127661; doi:10.3389/fgene.2025.1522338)
Supplement: Supplementary file 13 [file DataSheet1.docx]

## Supplementary Figures

## Supplementary Figures


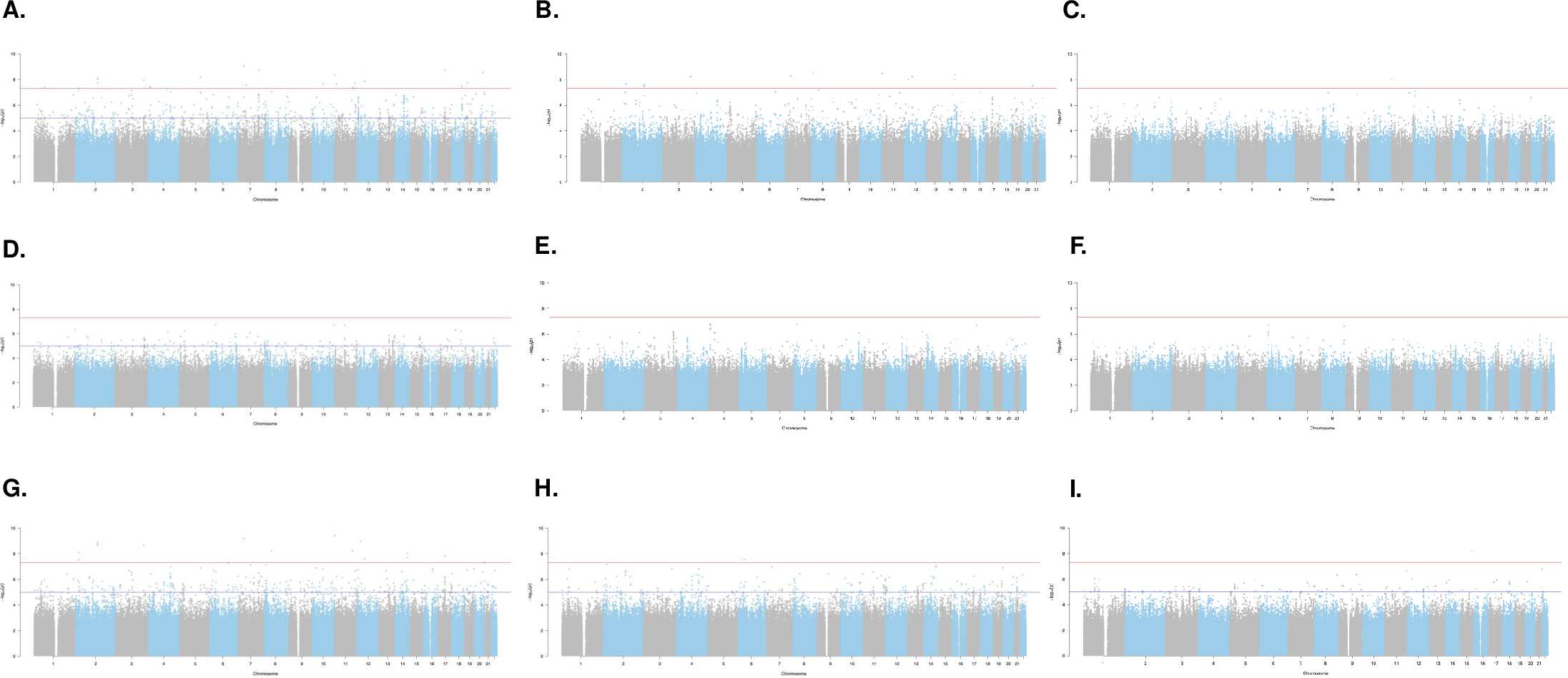


**Figure S1. GWAS on the Discovery sample: Manhattan plots for each NHF trait. A.** Manhattan plot relative to the 0.25 kHz trait**. B.** Manhattan plot relative to the 0.50 kHz trait. **C.** Manhattan plot relative to the 1 kHz trait. **D.** Manhattan plot relative to the 2 kHz trait. **E.** Manhattan plot relative to the 4 kHz trait. **F.** Manhattan plot relative to the 8 kHz trait. **G.** Manhattan plot relative to the PTAL trait. **H.** Manhattan plot relative to the PTAM trait. **I.** Manhattan plot relative to the PTAH trait. On the *x-axis* chromosomal position for each variant is reported. The y-axis shows the − log10 *p*-value. The genome-wide significance threshold (*p*-value = 5x10^-8^) is represented by the red horizontal line; the blue horizontal line represents the suggestive significance threshold (*p*-value = 1x10^-5^).

**
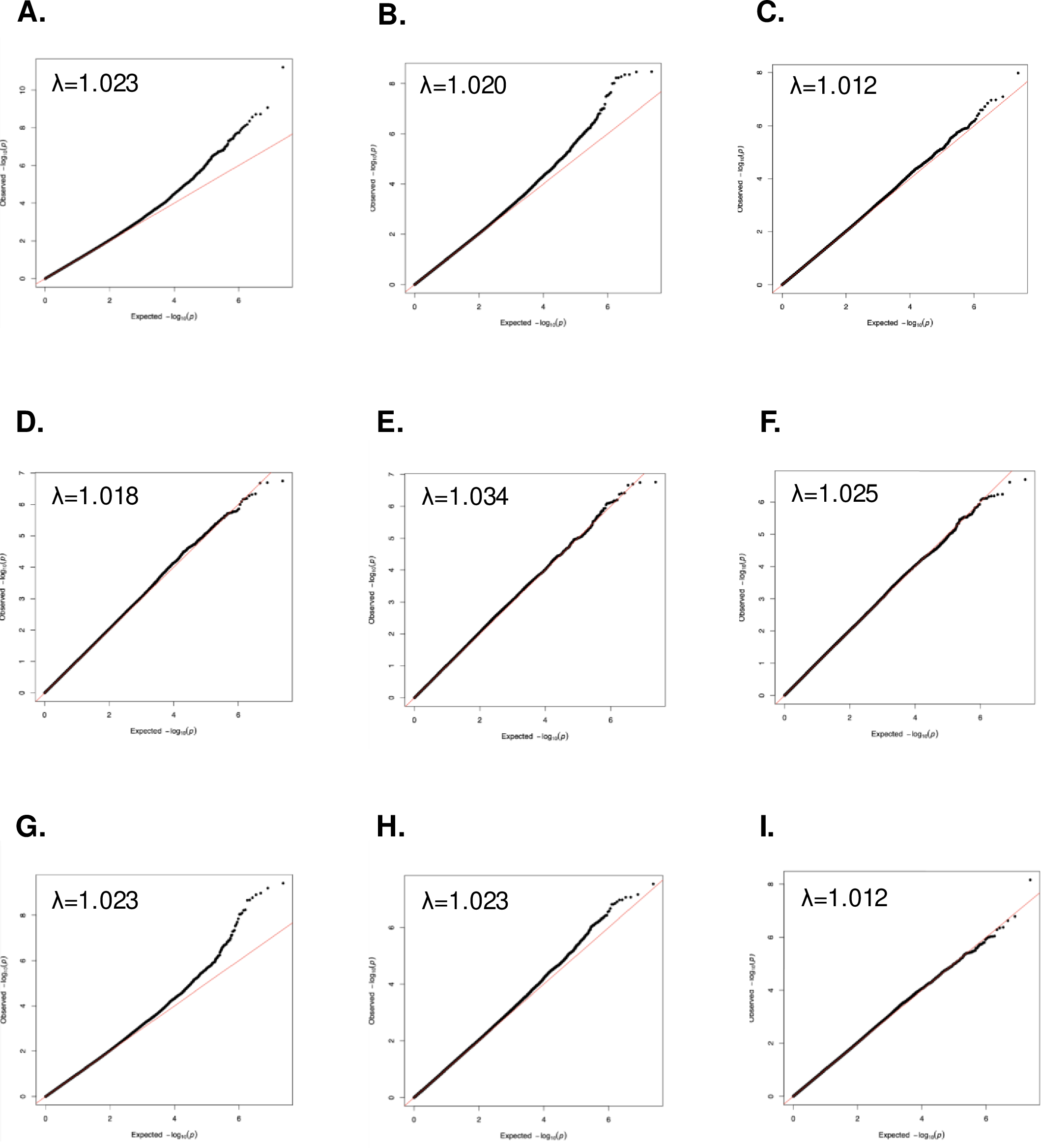
**

**Figure S2. GWAS on the Discovery sample: Q-Q plots for each NHF trait.** **A.** Q-Q plot relative to the 0.25 kHz trait. **B.** Q-Q plot relative to the 0.50 kHz trait. **C)** Q-Q plot relative to the 1 kHz trait. **D.** Q-Q plot relative to the 2 kHz trait. **E.** Q-Q plot relative to the 4 kHz trait. **F.** Q-Q plot relative to the 8 kHz trait. **G.** Q-Q plot relative to the PTAL trait. **H.** Q-Q plot relative to the PTAM trait. **I.** Q-Q plot relative to the PTAH trait.


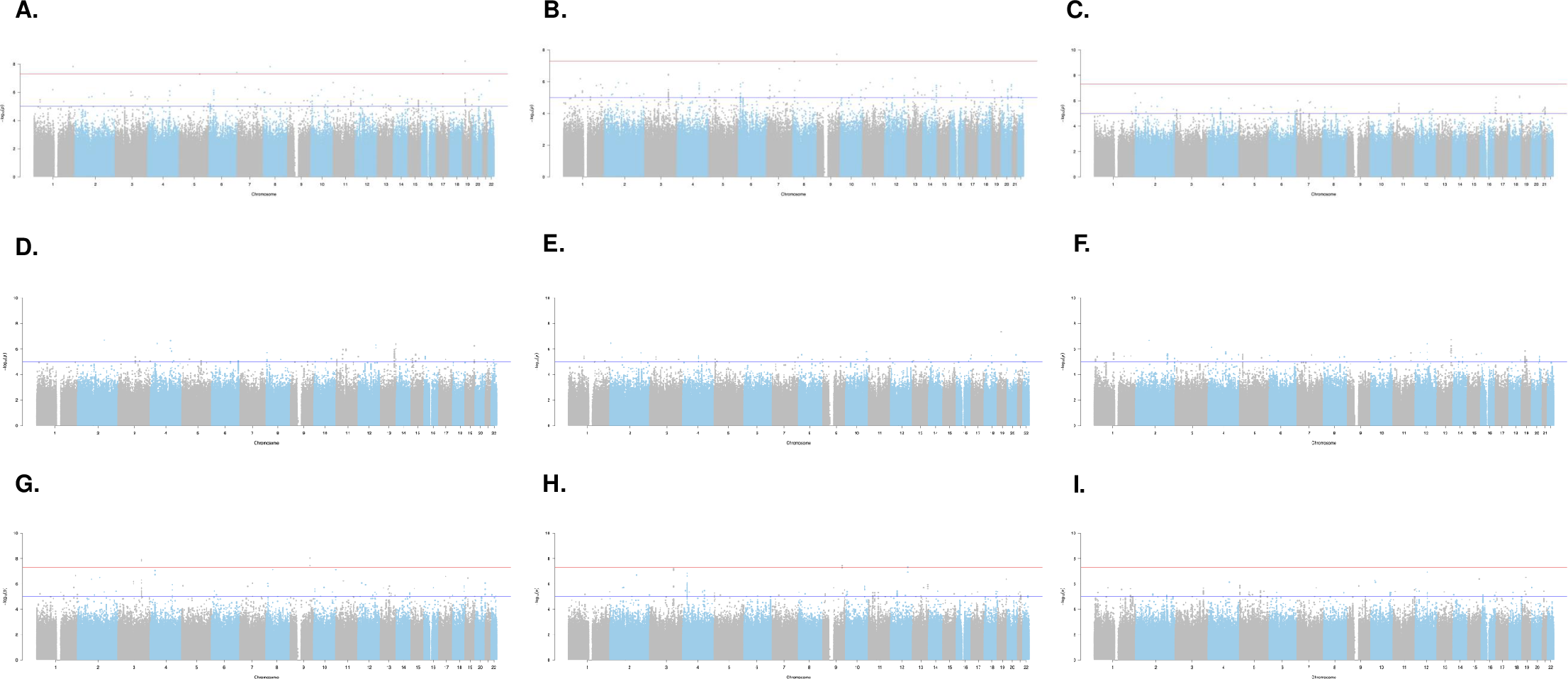


**Figure S3. GWAS Meta-analyses on the Combined sample: Manhattan plots for each NHF trait. A.** Manhattan plot relative to the 0.25 kHz trait**. B.** Manhattan plot relative to the 0.50 kHz trait. **C.** Manhattan plot relative to the 1 kHz trait. **D.** Manhattan plot relative to the 2 kHz trait. **E.** Manhattan plot relative to the 4 kHz trait. **F.** Manhattan plot relative to the 8 kHz trait. **G.** Manhattan plot relative to the PTAL trait. **H.** Manhattan plot relative to the PTAM trait. **I.** Manhattan plot relative to the PTAH trait. On the *x-axis* chromosomal position for each variant is reported. The y-axis shows the − log10 *p*-value. The genome-wide significance threshold (*p*-value = 5x10^-8^) is represented by the red horizontal line; the blue horizontal line represents the suggestive significance threshold (*p*-value = 1x10^-5^).


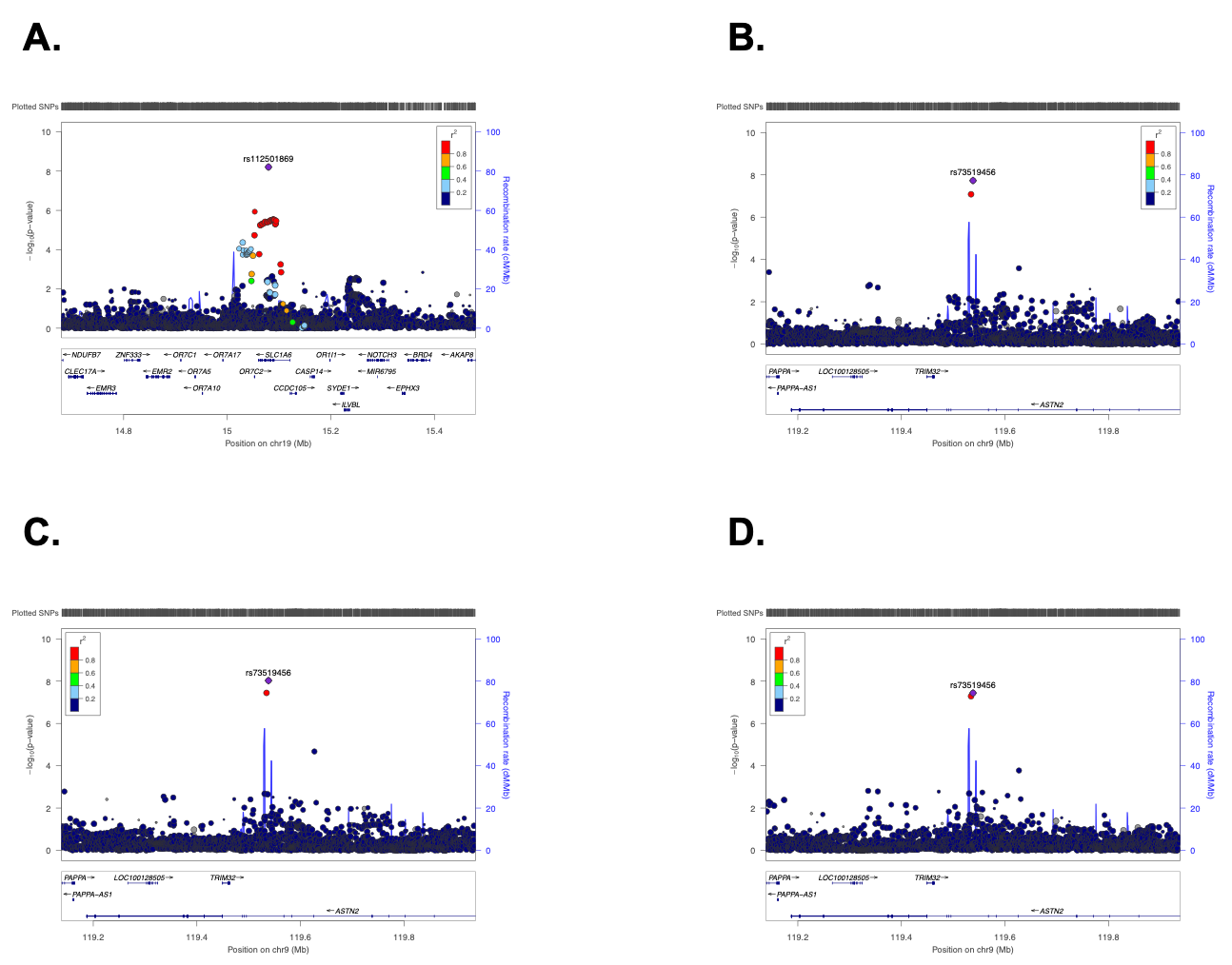


**Figure S4. Regional association plot for the genome-wide significant signals from the GWAS Meta-analyses. A.** Regional plot relative to chromosome 19 for the 250Hz trait. **B.** Regional plot relative to chromosome 9 for the 500Hz trait. **C.** Regional plot relative to chromosome 9 for the PTAL trait. **D.** Regional plot relative to chromosome 9 for the PTAM trait. The *y-axis* shows the minus logarithm of the p-value, while the *x-axis* represents the chromosomal position (with genes annotation). For each SNP, the Linkage Disequilibrium values are colour coded, with the lead SNP in violet. Plots were realized in LocusZoom (Pruim et al.,2010).


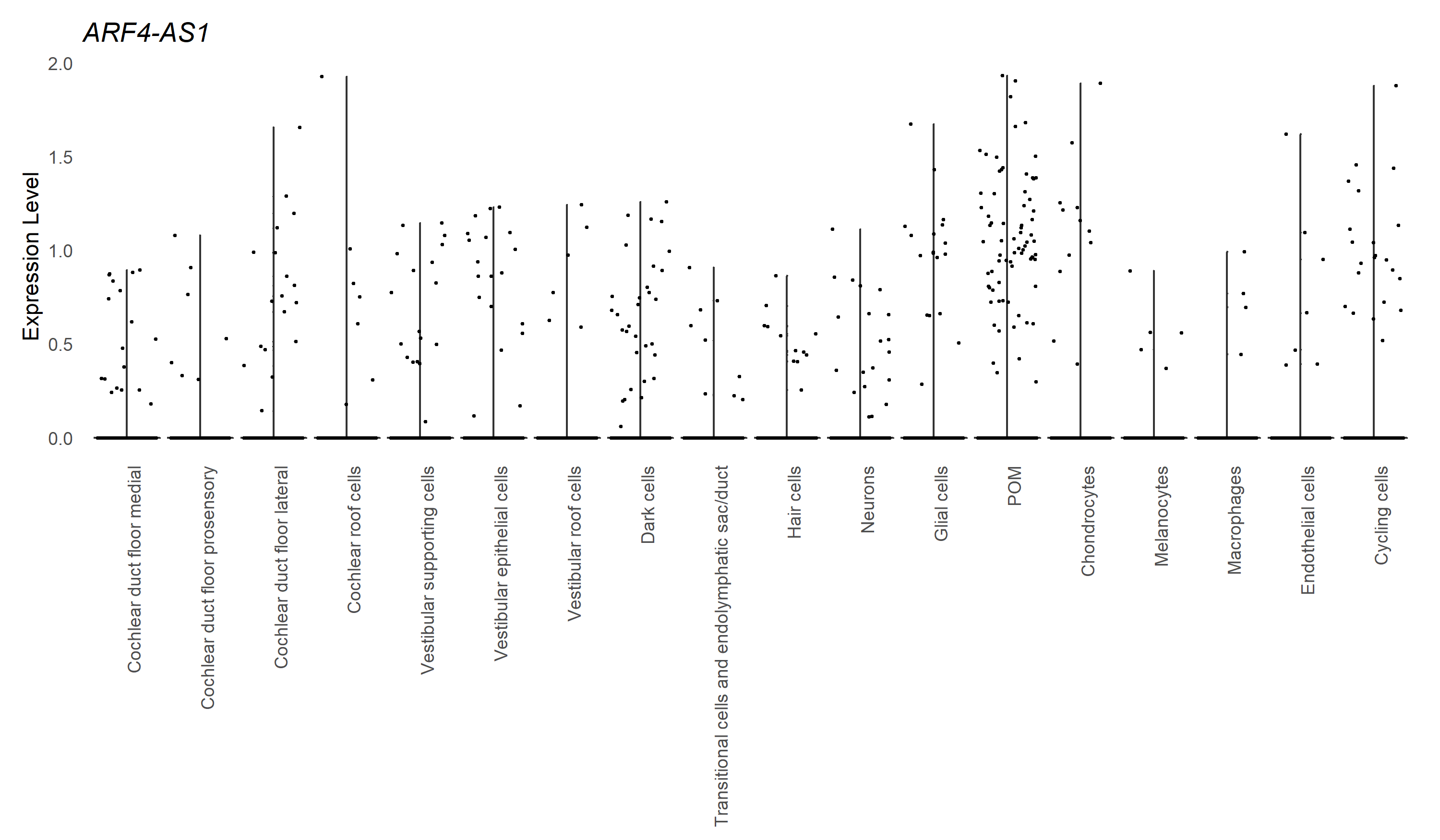


**Figure S5. Violin plots showing expression levels of genes prioritized in the TWAS analysis for the 0.25 kHz traits.** The violin plots report the expression levels of *ARF4-AS1* gene in human inner ear tissues, extracted from snRNA-seq data (doi: 10.1016/j.celrep.2023.112623). In the *x-axis* inner ear cell types are detailed, and in the *y-axis*, expression values are reported.
